# A Lightweight Multi-Mental Disorders Detection Method Using Entropy-Based Matrix from Single-Channel EEG Signals

**DOI:** 10.3390/brainsci14100987

**Published:** 2024-09-28

**Authors:** Jiawen Li, Guanyuan Feng, Jujian Lv, Yanmei Chen, Rongjun Chen, Fei Chen, Shuang Zhang, Mang-I Vai, Sio-Hang Pun, Peng-Un Mak

**Affiliations:** 1 School of Computer Science, Guangdong Polytechnic Normal University, Guangzhou 510665, China; lijiawen@gpnu.edu.cn (J.L.); fengguanyuan@gpnu.edu.cn (G.F.); chenyanmei@gpnu.edu.cn (Y.C.); chenrongjun@gpnu.edu.cn (R.C.); 2 Hubei Province Key Laboratory of Occupational Hazard Identification and Control, Wuhan University of Science and Technology, Wuhan 430065, China; 3 Guangdong Provincial Key Laboratory of Intellectual Property and Big Data, Guangdong Polytechnic Normal University, Guangzhou 510665, China; 4 Department of Electrical and Electronic Engineering, Southern University of Science and Technology, Shenzhen 518055, China; fchen@sustech.edu.cn; 5 School of Artificial Intelligence, Neijiang Normal University, Neijiang 641004, China; zhang.s@njtc.edu.cn; 6 School of Life Science and Technology, University of Electronic Science and Technology of China, Chengdu 610056, China; 7 Department of Electrical and Computer Engineering, University of Macau, Macau 999078, China; fstmiv@um.edu.mo (M.-I.V.); lodgepun@um.edu.mo (S.-H.P.); fstpum@um.edu.mo (P.-U.M.); 8 State Key Laboratory of Analog and Mixed-Signal VLSI, University of Macau, Macau 999078, China

**Keywords:** mental disorders detection, electroencephalography (EEG), entropy, machine learning

## Abstract

**Background**: Mental health issues are increasingly prominent worldwide, posing significant threats to patients and deeply affecting their families and social relationships. Traditional diagnostic methods are subjective and delayed, indicating the need for an objective and effective early diagnosis method. **Methods**: To this end, this paper proposes a lightweight detection method for multi-mental disorders with fewer data sources, aiming to improve diagnostic procedures and enable early patient detection. First, the proposed method takes Electroencephalography (EEG) signals as sources, acquires brain rhythms through Discrete Wavelet Decomposition (DWT), and extracts their approximate entropy, fuzzy entropy, permutation entropy, and sample entropy to establish the entropy-based matrix. Then, six kinds of conventional machine learning classifiers, including Support Vector Machine (SVM), k-Nearest Neighbors (kNN), Naive Bayes (NB), Generalized Additive Model (GAM), Linear Discriminant Analysis (LDA), and Decision Tree (DT), are adopted for the entropy-based matrix to achieve the detection task. Their performances are assessed by accuracy, sensitivity, specificity, and F1-score. Concerning these experiments, three public datasets of schizophrenia, epilepsy, and depression are utilized for method validation. **Results**: The analysis of the results from these datasets identifies the representative single-channel signals (schizophrenia: O1, epilepsy: F3, depression: O2), satisfying classification accuracies (88.10%, 75.47%, and 89.92%, respectively) with minimal input. **Conclusions**: Such performances are impressive when considering fewer data sources as a concern, which also improves the interpretability of the entropy features in EEG, providing a reliable detection approach for multi-mental disorders and advancing insights into their underlying mechanisms and pathological states.

## 1. Introduction

Currently, approximately 970 million people suffer from mental disorders, which usually lead to varying degrees of impairment in cognitive, emotional, and behavioral mental activities [1]. Typical mental disorders include schizophrenia, epilepsy, depression, and so on. Schizophrenia is a severe mental disorder marked by symptoms such as hallucinations, delusions, disordered thinking, erratic behavior, and agitation. Particularly, more devastating than other mental disorders, acute schizophrenia significantly reduces the life expectancy of those affected by nearly 20 years compared to the general population [2]. Second, epilepsy, a neurological condition caused by abnormal electrical discharges in the brain, results in episodes characterized by loss of consciousness and prolonged seizures, affecting about 50 million people globally [3]. Lastly, depression is a profoundly debilitating mental health disorder characterized by persistent sadness, self-doubt, and even severe suicidal tendencies [4]. Based on statistics from the World Health Organization (WHO), an estimated 4.4% of the global population, which equates to approximately 322 million people, are currently living with depression [5]. During the first year of the Coronavirus Disease 2019 (COVID-19) pandemic, the incidence of depression saw a significant rise of 25%. This increase translates to approximately 80 million additional cases of depression. Despite continuous updates to diagnostic instruments and treatment methods, the scarcity of medical equipment and outdated healthcare standards means that few patients are identified early and receive timely treatment. Hence, mental disorders are spreading progressively worldwide [6].

Undoubtedly, early detection of mental disorders is vital. However, their diagnosis relies heavily on the doctor’s experience of the patient’s symptoms and medical expertise, making it highly subjective. For example, in the diagnosis of depression, in addition to the use of self-rating scales from the *International Classification of Diseases*, 11th Edition (ICD-11) [7] and the *Diagnostic and Statistical Manual of Mental Disorders*, 5th Edition (DSM-5) [8], there are also traditional psychometric questionnaires, such as the Beck Depression Inventory (BDI) [9] and the Hamilton Depression Rating Scale (HDRS) [10]. According to a meta-analysis of 50,731 patients from 118 studies by Mitchell et al. [11], the accuracy of depression diagnosis was found to be only 47.3%. Depending on questionnaire self-assessment is a time-consuming and inaccurate approach, especially in populations with a weak sense of self-judgment (e.g., children and the elderly), and its accuracy plummets. Therefore, a reliable and objective detection way is preferred and desired in this field.

Electroencephalography (EEG) records electrophysiological signals of neuronal activity in the brain, providing an objective response to brain activity and playing a vital role in diagnosing mental disorders [12]. Specifically, it is a non-invasive method that only requires attaching electrodes to the scalp for collecting electrical signals, making it a safer option. In addition, it is capable of resolving the electrical activity of the brain at the millisecond level, offering the possibility of studying rapid changes in the brain. Thus, this technique is widely used not only in clinical diagnoses such as epilepsy, depression, schizophrenia, and so on but also in neuroscience, psychology, and cognitive science. For example, Movahed et al. [13] designed a classification framework for depression by extracting the statistical, frequency domain, and brain region association features of EEG, which can accomplish a classification accuracy of 99%. Qiao et al. [14] introduced a TanhReLU-based Convolutional Neural Network (CNN) for EEG-based classification of Major Depression Disorder (MMD), offering a promising accuracy of 98.59%. Gupta et al. [15] developed a privacy-preserving federated learning-based multimodal system utilizing Bidirectional Long Short-term Memory (BiLSTM) through audio and EEG data, reaching an accuracy of 99.9% for the MMD detection task. Hu et al. [16] presented an Iterative Gated Graph Convolutional Network (IGGCN) for epileptic seizure detection with an average F1-score and recall of 91.5%, and 91.8%, respectively. Nithya et al. [17] used a majority rule-based Local Binary Pattern (LBP) approach to achieve the highest accuracy of 95.18% on the Freiburg dataset for epileptic seizure detection. Baygin et al. [18] developed a hybrid deep learning network to extract the features of EEG to conduct autism spectrum disorder detection with an accuracy of 96.44%. Kumar et al. [19] extracted both Histogram of Local Variance (HLV) and Symmetrical Weighted Local Binary Pattern (SLBP) features from EEG signals for detecting schizophrenia, realizing an accuracy of 92.85%. Mardini et al. [20] adopted a Genetic Algorithm (GA) in conjunction with four models for EEG signal analysis for epilepsy detection. They found that an Artificial Neural Network (ANN) achieves a higher accuracy. Chen et al. [21] offered a short-time sequence model based on a CNN to extract features from EEG signals for building a detection framework for depression with an accuracy of 99.15%. In other fields, Wang et al. [22] combined time-frequency and non-linear features of EEG to classify bruxism using a fine-tree classifier. Similarly, Bardak et al. [23] proposed a model consisting of Adaptively Designed Neuro-fuzzy Inference System (ANFIS) classifiers in parallel, obtaining great results in emotion recognition utilizing EEG signals. These aforementioned studies demonstrate that EEG is an effective input data source for mental disorders detection and other related fields, whether employing traditional machine learning methods or deep learning models.

Generally, an EEG-based mental disorders detection framework consists of signal processing, feature extraction, and classification model establishment. First, because EEG signal acquisition is susceptible to noise from sources such as eye movements, blinking, cardiac activity, and muscle movements, it is necessary to filter them for obtaining pure EEG data. To this end, a bandpass filter and a fourth-order Butterworth filter can eliminate both the high-frequency noise and low-frequency artifacts [24]. In another study by Wirsich et al. [25], filters were used not only to control the frequency contents between 0.5 Hz and 70 Hz but also to eliminate the power supply frequency noise generated during the acquisition process. Then, to further analyze the specificity of the EEG signals and provide quality inputs to the classifier, trustworthy features need to be extracted, which are typically categorized into the statistical domain (e.g., mean, skewness, kurtosis, maximum, minimum, empirical distribution function percentile, empirical distribution function slope, etc.), spectral domain (e.g., Fast Fourier Transform (FFT), Wavelet Transform (WT), spectral fundamental frequency, spectral maximal peak, etc.), and temporal domain (e.g., auto-correlation, differential mean, curve coverage area, cross-collar rate, etc.) [26]. In addition, deep learning models such as CNN can be utilized to implement extractors for extracting valuable features automatically [27]. Finally, conventional machine learning classifiers, such as Support Vector Machine (SVM), k-Nearest Neighbors (kNN), Naive Bayes (NB), Generalized Additive Model (GAM), Linear Discriminant Analysis (LDA), Decision Tree (DT), etc., are employed to categorize EEG signals based on applied features [28]. Moreover, diverse neural network models, such as LSTM, ANN, Recurrent Neural Network (RNN), and Temporal Convolutional Network (TCN), are also available [29]. Meanwhile, multiple classifiers can be integrated using ensemble learning methods, such as bagging, boosting, and random subspace, to further enhance the performance of hybrid classification models [30]. As seen, the previous mental disorders detection contributes to the field of brain science. Nonetheless, most of them are only specific to one kind of disorder (e.g., schizophrenia, epilepsy, depression, or others), and an approach that is well-suited for various mental disorders is limited.

**Figure 1:**
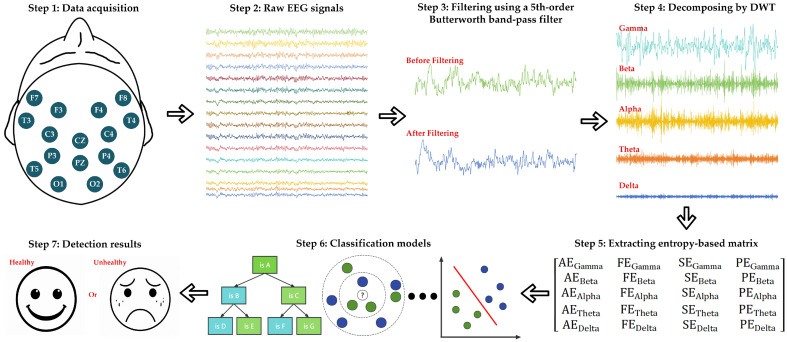
The overall framework of the proposed lightweight mental disorders detection method.

To address this drawback, this paper proposes a lightweight detection method for multi-mental disorders employing the entropy-based matrix derived from single-channel EEG signals, which offer non-invasive and real-time monitoring of brain activity. This paper aims to address the critical need for objective and effective early detection methods for mental disorders, particularly in light of the limitations of traditional subjective diagnostic tools. Thus, the significance of this paper lies in its potential to enhance diagnostic accuracy with fewer data sources, making the approach not only more accessible but also portable, enabling broad applicability in healthcare cases. To achieve this, first, it is necessary to filter the interference of noisy signals, and a fifth-order Butterworth filter is applied to obtain the information within the frequency range of 0.5–70 Hz of the EEG recording. Second, since the abnormal activities of Delta (δ), Theta (θ), Alpha (α), Beta (β), and Gamma (γ) waves are related to mental disorders, the Discrete Wavelet Transform (DWT) is applied to decompose the EEG signals into those aforementioned waves that represent the frequency ranges of 0.5–4 Hz, 4–8 Hz, 8–16 Hz, 16–32 Hz, and 32–64 Hz, respectively, which are also known as brain rhythms. Next, to better quantify the abnormal activities of five brain rhythms, the approximate entropy (AE), fuzzy entropy (FE), sample entropy (SE), and permutation entropy (PE) are extracted from single-channel EEG signals, so 20 features in total for one channel. Such entropy features describe the signal instability, where AE evaluates the irregularity of the signal, FE measures noise and uncertainty better, improving recognition of complex signals, SE quantifies the complexity and randomness of the signal, and PE captures the non-linear characteristics of the signal. Subsequently, to characterize the signal comprehensively, these 20 features are applied to generate an entropy-based matrix, providing quality inputs for the machine learning classifiers. Finally, six conventional classifiers, including SVM, kNN, NB, GAM, LDA, and DT, are employed for the entropy-based matrix to investigate the detection tasks for three public datasets of schizophrenia [31], epilepsy [32], and depression [33]. In addition, to avoid overfitting in the limited experimental samples in the datasets, leave-one-out cross-validation (LOOCV) is used to evaluate classification performance. After the analysis of the results from three datasets, the representative single-channel signals, as well as the optimal classifiers, can be identified. Such findings reliably assess the validity of the proposed method and ensure that its results maintain robustness for individuals with various mental disorders. For better illustration, the overall framework is depicted in Figure 1.

Particularly, this paper provides the following contributions:

1. A multi-mental disorders detection method based on the entropy-based matrix is proposed, which not only increases the interpretability of entropy features to detect the abnormal activity of brain rhythms but also offers a reliable solution for various mental disorders;
2. From the experimental results, both the optimal classifier with high generalizability and the representative channel with impressive classification performance are selected. Such a lightweight way provides the proper classifiers and channels that are beneficial for developing portable mental disorder detection devices through few data sources;
3. The method validation employs three mental disorders datasets (schizophrenia, epilepsy, and depression), helping to advance insights into the underlying mechanisms and pathological states of these disorders with great detail.

The rest of this paper is organized as follows: Section 2 presents the experimental datasets. Then, the details about the proposed multi-mental disorders detection method are described in Section 3. Section 4 shows the results and discussion. Finally, the conclusion is drawn in Section 5.

## 2. Experimental Datasets

**Figure 2:**
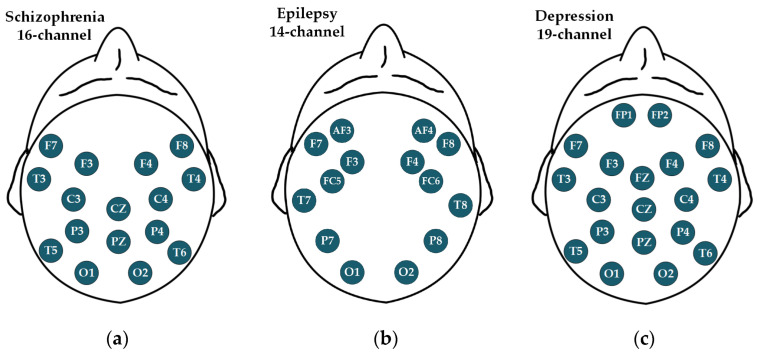
EEG channels of the three mental disorders datasets: (**a**) schizophrenia, 16-channel; (**b**) epilepsy, 14-channel; and (**c**) depression, 19-channel.

Aiming to a multi-mental disorders detection method, the EEG signals from diverse conditions are necessary for evaluations. Consequently, three typical mental disorders, schizophrenia [31], epilepsy [32], and depression [33], have been investigated. Figure 2 draws their corresponding EEG channels, and Table 1 summarizes their key information. More details are described as follows:

### 2.1. Schizophrenia

The schizophrenia dataset [31] is a publicly available resource from the Laboratory of Neurophysiology and Neurocomputer Interfaces at Moscow State University. During data collection, the subjects were divided into two groups after observation by professional doctors of the Psychiatric Research Center: one group of healthy school adolescents (39 subjects) and the other group of adolescents with symptoms of schizophrenia (45 subjects). EEG signals of a 1 min duration were acquired from both groups of subjects using a 16-channel device (shown in Figure 2a) with a sampling frequency of 128 Hz.

### 2.2. Epilepsy

The epilepsy dataset [32] was collected in two regions of Africa: Guinea-Bissau and Nigeria. In Guinea-Bissau, 97 subjects participated, including 51 epileptic patients and 46 healthy controls. Each subject participated in approximately five minutes of EEG signal collection, during which the first three minutes were recorded with eyes closed and the subsequent two minutes with eyes open, resulting in the collection of 97 sets of EEG signals. In Nigeria, 221 subjects participated, including 128 epileptic patients and 93 healthy controls. Subjects were numbered in order of participation, with odd-numbered subjects tested with eyes open and even-numbered subjects tested with eyes closed, resulting in 221 sets of EEG signals. In total, 179 sets of epileptic signals and 139 sets of healthy signals. In addition, the experiments were conducted using a 14-channel data acquisition device (shown in Figure 2b) with a sampling frequency of 128 Hz.

### 2.3. Depression

The depression dataset [33] was acquired from an experiment approved by the Human Ethics Committee of Hospital Universiti Kebangsaan Malaysia, Kelantan, Malaysia. The experiment recruited two groups: one group of 34 depression outpatients (17 males and 17 females, aged between 27 and 53 years old) and another group of 30 age-matched healthy controls (21 males and 9 females) without any other mental disorders. Both groups were diagnosed by a medical professional to ensure that their conditions matched the subgroup category they were placed in. Then, EEG signals were recorded in two states, eyes-open and eyes-closed, resulting in 128 sets of EEG signals. In addition, the signals were collected utilizing an international standard 10–20 system (19-channel, shown in Figure 2c) with a sampling frequency of 256 Hz. Since the eye state of the subject is not the aim of this paper, the signals from both eyes-open and eyes-closed states were mixed for the experiment. Moreover, several signals were not provided on the public website due to data loss, resulting in 119 sets of EEG signals (57 sets for depression and 62 sets for healthy controls) for method evaluation.

## 3. Proposed Method

### 3.1. Signal Processing

First, as raw EEG signals are often contaminated by various artifacts and noise, such as Electrocardiography (ECG) and Electrooculography (EOG), a preprocessing step is necessary to enhance the signal quality and reliability of the analysis. Thus, the raw EEG signals are filtered through a fifth-order Butterworth band-pass filter to obtain the 0.5–70 Hz portion, effectively removing those noisy interferences and ensuring the signal is reliable for further analysis. Once filtered, the EEG signals are transformed from the time domain to the frequency domain for decomposition. Since the FFT is limited in acquiring frequency information that changes over time, DWT is employed, which enriches the concept of localization by providing a dynamic time-frequency window that adapts to frequency changes. This adaptability can help avoid the data redundancy that arises from the Continuous Wavelet Transform (CWT) [34]. In addition, the orthogonality of the Daubechies 4 (DB-4) wavelet basis function enhances the efficiency of signal processing, as its smoothness makes it highly suitable for analyzing smooth transitions and detailed variations, which is beneficial for EEG signals containing physiological brain information and subtle fluctuations [35].

**Figure 3:**
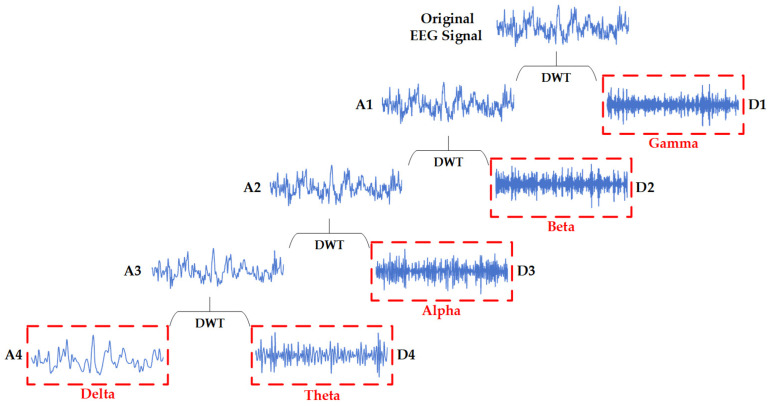
The 4-level DWT of EEG signals with DB-4 as basis wavelet function.

DWT can be represented as a binary tree implemented by a multi-layered set of high and low-frequency filters. Depending on the sampling rate of the datasets used, different levels of DWT are required for decomposition. For example, an EEG signal *x*[*t*] with a sampling rate of 128 Hz needs to be decomposed by a four-level DWT through DB-4 as the basis wavelet function, as illustrated in Figure 3.

In Figure 3, for the first level of decomposition, *x*[*t*] is divided into a high-frequency coefficient (D1) and a low-frequency coefficient (A1). In the second level, A1 is further decomposed into D2 and A2, and this process continues. Specifically, A4, D4, D3, D2, and D1 correspond to five brain rhythms in the proposed method, i.e., Delta, Theta, Alpha, Beta, and Gamma waves, respectively. This decomposition allows for a detailed analysis of the EEG signals, acquiring the essential characteristics of such brain rhythms.

Typically, the DWT is implemented through Multi-Resolution Analysis (MRA) and filter banks. As for the MRA, the input signal *x*[*t*] is decomposed into low-frequency and high-frequency components at different scales. The low-frequency component, known as the approximation coefficient, indicates the coarse information of the signal. The high-frequency component, known as the detail coefficient, contains the fine details of the signal. The filter banks consist of a low-pass filter for extracting the approximation coefficients and a high-pass filter for obtaining the detail coefficients, as expressed in Equations (1) and (2):

(1) $$a_{j+1}[k]=\sum_{n}h[n-2k]a_{j}[n]$$

(2) $$d_{j+1}[k]=\sum_{n}g[n-2k]d_{j}[n]$$

where *a_j_* represents the level-*j* approximation coefficient, *d_j_* denotes the level-*j* detail coefficient, *h*[*n*] means the low-pass filter, *g*[*n*] is the high-pass filter, *n* denotes the index of the discrete sample point of the current level signal, and *k* means the index of wavelet coefficients after downsampling.

When employing the DB-4 as a basis wavelet function of DWT, its low-pass filter coefficients and high-pass filter coefficients are shown in Equations (3) and (4):

(3) $$h[0]=\frac{1+\sqrt{3}}{4\sqrt{2}},h[1]=\frac{1+\sqrt{3}}{4\sqrt{2}},h[2]=\frac{1+\sqrt{3}}{4\sqrt{2}},h[3]=\frac{1+\sqrt{3}}{4\sqrt{2}}$$

(4) $$g[n]={\left(-1\right)}^{n}h[3-n]$$

### 3.2. Feature Extraction

The second step is feature extraction, which can be generally divided into two main categories [36]. One involves extracting features using a feature extractor constructed by deep learning models. While this approach beneficially enhances classification accuracy, it necessitates a large number of samples for training the model. Another way employs statistical, non-linear, functional connectivity, or other features to detect mental disorders. Although the DWT is also utilized to analyze the spectral domain, it typically entails energy analysis or estimating the power spectral density. In this regard, entropy analysis of dynamics reveals vital information often not captured in spectral analysis and is well-suited for analyzing non-stationary signals [37]. That means the entropy analysis is sensitive to non-linear dynamics and less affected by artifacts and noise. Specifically, each of these entropy measures exhibits properties that complement one another in quantifying the non-linear dynamics of brain activity. As stated, AE is sensitive to signal irregularity, SE addresses bias in small datasets, FE handles noise effectively, and PE captures the non-linear time series characteristics. This combination allows for a robust representation of the brain rhythms across different states. Therefore, four kinds of entropies, i.e., AE, FE, SE, and PE, are extracted to analyze and characterize brain rhythms in the proposed method. This step can assess the irregularity, complexity, and randomness of EEG, enabling the identification of abnormal brain activities and the classification of mental disorders accordingly.

AE quantifies the regularity and unpredictability of fluctuations in time-series data, which require only a relatively short amount of data to produce a more robust estimate and is suitable for characterizing non-stationary, non-linear EEG signals. Mathematically, AE is obtained as follows [38]:

Suppose that a time series containing *N* data points: u(1),u(2),…,u(N). Construct it as a subsequence of length *m*: $\vec{x}\left(1\right)$,$\vec{x}\left(2\right)$,…,$\vec{x}\left(N-m+1\right)$, where $\vec{x}\left(i\right)=\left\{u(i),u\left(i+1\right),\ldots ,u\left(i+m-1\right)\right\},i=1\to N-m+1$.

First, the maximum distance $d[\vec{x}\left(i\right),\vec{x}(j)]$ between each $\vec{x}\left(i\right)$ and $\vec{x}(j)$ from all other subsequences is calculated by Equation (5):

(5) $$d\left[\vec{x}\left(i\right),\vec{x}\left(j\right)\right]={max}_{k=0\to m-1}\left|\vec{x}(i+k)-\vec{x}(j+k)\right|$$

Then, the number of cases where $d[\vec{x}\left(i\right),\vec{x}(j)]$ is less than r (threshold) is counted and its ratio to the total number of N−m is calculated and denoted as $C_{i}^{m}(r)$:

(6) $$C_{i}^{m}\left(r\right)=\frac{1}{N-m}\left\{d\left[\vec{x}\left(i\right),\vec{x}\left(j\right)\right]<r\right\}$$

Next, the logarithm of $C_{i}^{m}(r)$ is obtained, and the average of all the values taken by *i* is calculated and denoted as $\phi^{m}(r)$:

(7) $$\phi^{m}(r)=\frac{1}{N-m+1}\sum_{i=1}^{N-m+1}lnC_{i}^{m}(r)$$

Lastly, by repeating the above steps, the AE value is acquired:

(8) $$\mathrm{AE}(m,r)={lim}_{N\to \infty }[\phi^{m}(r)-\phi^{m+1}(r)]$$

SE is similar to the AE calculation process but excludes *i* = *j* cases when traversing all combinations of $\vec{x}\left(i\right)$ and $\vec{x}\left(j\right)$, which is insensitive to lost data, even if as much as 1/3 of data are lost [39].

FE indicates the complexity and uncertainty of time-series data. Unlike other entropy features, it employs fuzzy set theory to represent similarities in time series as fuzzy affiliations. This way avoids the loss of information due to binary classification, providing a more stable and continuous measure of complexity. Its value is calculated as follows [40]:

Similar to the AE, but with a slightly different presence in $\vec{x}\left(i\right)$, where $\vec{x}\left(i\right)=\{\{x[(u(i)],x[(u(i+1))],\ldots ,x[(u(i+m-1))]\}-x_{0}[u(i)]\},i=1\to N-m+1,$ and $x_{0}[u(i)]$ is the mean of the consecutive $\vec{x}\left(i\right)$:

(9) $$x_{0}\left(i\right)=\frac{1}{m}\sum_{k=0}^{m-1}x(i+k)$$

Like (5), the distance between each $\vec{x}\left(i\right)$ and $\vec{x}\left(j\right)$ from all other subsequences is calculated at first, excluding the case of *i* = *j*. Then, a new variable named fuzzy weight is applied. Combining with the threshold *r* and the distance $d[\vec{x}\left(i\right),\vec{x}(j)]$ to measure the similarity between $\vec{x}\left(i\right)$ and $\vec{x}\left(j\right)$, denoted as $D_{ij}^{m}$:

(10) $$D_{ij}^{m}(n,r)=e^{-{\left(d_{ij}^{m}\right)}^{n}/r}$$

Next, to define a new function called $\phi^{m}(n,r)$:

(11) $$\phi^{m}(n,r)=\frac{1}{N-m}\sum_{i=1}^{N-m}\left[\frac{1}{N-m-1}\sum_{j=1,j\neq i}^{N-m}D_{ij}^{m}(n,r)\right]$$

As a result, the FE value is acquired by repeating the above steps:

(12) $$FE(m,n,r)={lim}_{N\to \infty }[\phi^{m}(n,r)-\phi^{m+1}(n,r)]$$

PE is useful for detecting kinetic mutations and time-series randomness and enables quantitative assessment of the random noise contained in the signals. It is appropriate for short-time data and has a certain degree of noise immunity, which makes it ideal for portable EEG-based systems. Its calculation can be expressed as follows [41]:

To perform phase space reconstruction on time-series *u* of length *N*, a matrix *Y* can be obtained:

(13) $$Y=\left[\begin{array}{ccc} \begin{array}{cc} x(1) & x(1+t)\\ x(2) & x(2+t)\\ x(j) & x(j+t) \end{array} & \begin{array}{c} \ldots \\ \ldots \\ \ldots \end{array} & \begin{array}{c} x(1+(m-1)t)\\ x(2+(m-1)t)\\ x(j+(m-1)t) \end{array}\\ \begin{array}{ccc} \vdots & & \vdots \end{array} & \vdots & \vdots \\ \begin{array}{cc} x(K) & x(K+t) \end{array} & \ldots & x(K+(m-1)t) \end{array}\right]$$

where *m* represents the embedding dimension, *t* is the delay time, and K=N−(m−1)t, so each row in *Y* represents a reconstructed component (totally *K*).

After that, rearranging each reconstructed component in ascending order yields a set of symbol sequences formed by the indexes of the positions of the elements in the vector, denoted as *S*(*l*):

(14) $$S\left(l\right)=\left\{j_{1},j_{2},\ldots ,j_{m}\right\},\;l=1,2,3,\ldots ,K$$

where *j*_1_, *j*_2_, …, *j_m_* represent the indices of the positions of the elements. So, by counting the number of occurrences of each *S*(*l*), the PE value is acquired through the probability of the *S*(*l*), denoted as *P_j_*:

(15) $$PE\left(u\right)=-\sum_{j=1}^{k}P_{j}\ln\left(P_{j}\right)$$

Here, from a preliminary test, set *m* = 2, *n* = 2, *r* = 0.15 times the standard deviation of the EEG signals.

### 3.3. Entropy-Based Matrix

After feature extraction, considering that abnormal brain rhythm activity is usually associated with mental disorders, the entropy features properly reveal the irregularity, complexity, randomness, and non-linearity of signals, which can further characterize these abnormal activities. Specifically, higher entropy values indicate more chaotic and complex signals, signifying more pronounced abnormal activities. Based on that, the proposed method uses the concept of feature-level fusion to generate an entropy-based matrix that consists of 20 features through the AE, SE, FE, and PE extracted from the five brain rhythms, as expressed in Equation (16):

(16) $$Entropy\;matirx=\left[\begin{array}{ccc} {AE}_{Gamma} & {FE}_{Gamma} & \begin{array}{cc} {SE}_{Gamma} & {PE}_{Gamma} \end{array}\\ {AE}_{Beta} & {FE}_{Beta} & \begin{array}{cc} {SE}_{Beta} & {PE}_{Beta} \end{array}\\ \begin{array}{c} {AE}_{Alpha}\\ \begin{array}{c} {AE}_{Theta}\\ {AE}_{Delta} \end{array} \end{array} & \begin{array}{c} {FE}_{Alpha}\\ \begin{array}{c} {FE}_{Theta}\\ {FE}_{Delta} \end{array} \end{array} & \begin{array}{cc} \begin{array}{c} {SE}_{Alpha}\\ \begin{array}{c} {SE}_{Theta}\\ {SE}_{Delta} \end{array} \end{array} & \begin{array}{c} {PE}_{Alpha}\\ \begin{array}{c} {PE}_{Theta}\\ {PE}_{Delta} \end{array} \end{array} \end{array} \end{array}\right]$$

The entropy-based matrix integrates five brain rhythms with four entropy features, effectively using the properties of rhythms in mental disorders and the sensitivity of entropies to describe non-stationary EEG signals. This fusion not only addresses the data insufficiency inherent in single-channel data but also facilitates a comprehensive analysis of the complex dynamic changes in EEG signals, enhancing the accuracy and interpretability of mental disorder detection. Therefore, the next step is utilizing the entropy-based matrix generated from each EEG signal as the input to train the classifiers and establish an appropriate classification model with the representative channel data.

### 3.4. Classification Method

Recent studies have shown that employing neural networks for the detection of mental disorders yields good performance. For instance, in [42], a combination of CNN and LSTM was used to achieve depression detection with an accuracy of 95.1%. In the same case, the accuracies through SVM, kNN, and DT classifiers were only 72.05%, 79.7%, and 79.49%, respectively. Nonetheless, as stated by Tawhid et al. [43], because neural networks generally require a large amount of training data and higher computational cost, the EEG signals are sliced to increase the amount of data for training. Yet, there will be a potential issue that arises when a segment of an EEG signal from a diseased one shows normal manifestations but is still labeled as diseased, which could lead to label confusion that reduces the reliability of the detection framework.

In addition, the number of features, i.e., the entropy-based matrix, in this paper is not large enough to satisfy the neural networks. Selecting the six conventional machine learning classifiers, including SVM, kNN, NB, GAM, LDA, and DT to evaluate a diverse set of models with varying strengths, is considered. In this paper, SVM is chosen for its well-known ability to handle non-linear data, particularly with the use of kernel methods. kNN is included due to its simplicity and effectiveness in smaller datasets. NB is selected because of its efficiency and relatively good performance with independent features. GAM and LDA are chosen as they offer strong interpretability and handle linear relationships well, making them useful for comparing against more complex models. Lastly, DT is selected for its ability to model non-linear relationships and handle noisy data. Overall, this selection covers a broad spectrum of model complexities, from simple models to more advanced ones, ensuring a comprehensive analysis of classifier performance on the entropy-based matrix.

Moreover, in place of an ablation study, which is commonly used in deep learning models, a comprehensive comparison of six conventional machine learning classifiers is conducted. Such a comparison helps to evaluate the contribution of each classifier to the detection task and determine the one with the highest performance and robustness. After that, the one that offers the satisfying performance is determined and included in the detection framework with the entropy-based matrix from the representative channel. The details of each classifier applied are described as follows:

SVM is a binary classification method that operates by finding a hyperplane that maximizes the margin between different classes. The key aspect of SVM is identifying the hyperplane that maximizes this margin, which can enhance the generalization ability of the classifier [44]. In this paper, a polynomial kernel is considered, specifically using the polynomial kernel function to map the data into a higher-dimensional space, which allows for the handling of non-linearly separable problems. In addition, to reduce model complexity and mitigate the risk of overfitting, a third-order polynomial kernel function is adopted. While SVM has limitations, such as potential unsuitability for large-scale datasets, this limitation may be advantageous given the relatively small dataset assessed in this paper.

kNN is an instance-based learning method used for classification. For an unknown sample, kNN identifies the k-Nearest Neighbors in the training set and utilizes their labels to vote on the class of the unknown sample [45]. In this study, k is set to 5, striking a balance between bias and variance, enhancing the robustness of the classification results. kNN is easy to implement. However, as the dataset grows, the computational complexity of kNN also increases, which is a limiting factor.

NB is a probabilistic classification method based on Bayes’ theorem, which assumes independence between features [46]. Although this assumption rarely holds in real-world scenarios, the NB classifier remains highly effective in many applications due to its simplicity and efficiency. In this paper, multivariate multinomial is chosen as the distribution type, which reflects the class distribution in the data and helps reduce model bias.

GAM is a classification method that captures complex non-linear relationships by representing the effects of features as the sum of multiple smoothing functions. The smoothing term is the core component of GAM, enabling it to obtain the non-linear relationships between features and response variables [47]. While GAM has limitations, such as sensitivity to both the model and the form of the smoothing function.

LDA is a linear classification method for modeling and prediction based on specific feature values. It seeks linear combinations of features that maximize between-class variance while minimizing within-class variance, making it a powerful tool for distinguishing between different classes [48]. The main advantages of LDA are computationally efficient and suitable for most classification tasks, as it can estimate the probabilities based on class frequencies, allowing the model to adapt well to varying datasets.

Lastly, DT employs a tree-like structure for classification. It partitions data through a series of decisions to achieve predictive outcomes. In classification tasks, DT assigns target variables based on the values of different features [49]. This paper uses the fitctree function to train the DT, utilizing the Gini impurity as the measure for evaluating splits. Each split in the tree is determined by selecting the feature that maximally reduces Gini impurity, effectively detecting abnormal brain activities.

Overall, each classifier has its advantages, making it essential to select the most appropriate one. To ensure the performance and generalizability of the proposed method, proper validation is necessary. While datasets are typically divided into training, validation, and test sets when sufficient data are available, this study utilizes three datasets with limited samples and pronounced individual specificity. To address these challenges, LOOCV is employed, which is particularly suitable for small datasets. In this approach, each subject’s data (i.e., entropy-based matrix extracted from the EEG signals of each subject) serves as the test set, while the data from the remaining subjects constitute the training set. This method maximizes the use of all available data for training and validation, reducing the impact of individual differences on model performance. It ensures that the classification model is not unduly influenced by specific data characteristics, aligning more closely with real-world scenarios where individuals use the proposed framework for mental disorder detection independently. Hence, the cross-individual performance validation effectively mitigates overfitting and enhances the model’s reliability. In addition, regarding the method validation, four evaluation metrics, including accuracy (ACC), sensitivity (SEN), specificity (SPE), and F1-score (F1), are applied in this study, as detailed in Equations (17)–(20):

(17) $$ACC=\frac{TN+TP}{TN+FN+TP+FP}$$

(18) $$SEN=\frac{TP}{FN+TP}$$

(19) $$SPE=\frac{TN}{FP+TN}$$

(20) $$F1=\frac{2\times TP}{2\times TP+FP+FN}$$

where True Positive (TP) means correctly predicted positive instances, True Negative (TN) denotes correctly predicted negative instances, False Negative (FN) represents incorrectly predicted negative instances (actual positives classified as negative), and False Positive (FP) indicates incorrectly predicted positive instances (actual negatives classified as positive).

## 4. Results and Discussion

In this experiment, the three publicly available EEG datasets are used to validate the performance of the proposed method based on MATLAB. To facilitate reproducible research and have a positive effect on the academic field, the source codes are freely available at https://github.com/75Fleven/CODE.git (accessed on 31 August 2024). In addition, to identify the minimum required number of channels (i.e., single-channel EEG signals) and the suitable classifier, a statistical analysis of performances across these datasets is performed according to the four evaluation metrics. Since the data properties of such mental disorders may differ, the results and discussion are presented based on each dataset.

In addition, an overall metric aimed at quantifying the process of optimal channel selection is designed. Given the practical significance of ACC, SEN, SPE, and F1, higher values in these metrics indicate better performance. For example, a higher SEN reflects a lower risk of missed diagnoses. Based on these considerations, the following steps are adopted to select the representative channel: Each metric is first ranked individually in descending order, meaning the higher the value, the higher the rank, with rankings represented by 1, 2, 3, …, N. Then, the rankings for the four metrics are summed to obtain a composite ranking, where a lower composite ranking indicates better overall performance within the proposed method. Thus, the channel with the lowest composite ranking is selected, which ensures that the representative channel of each mental disorder can be consistently identified.

### 4.1. Schizophrenia Results

**Figure 4:**
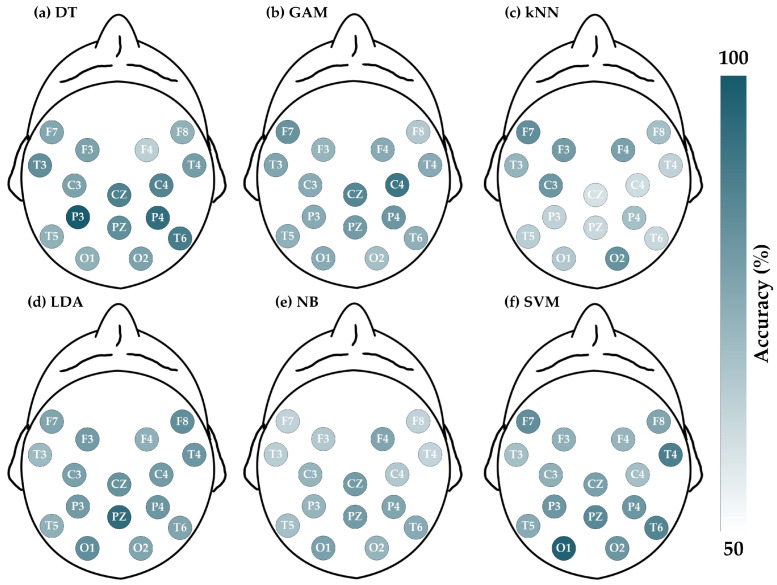
Evaluations of accuracy for schizophrenia dataset by entropy-based matrix generated from 16 channels using various classifiers. The deeper the color, the greater the accuracy: (**a**) DT; (**b**) GAM; (**c**) kNN; (**d**) LDA; (**e**) NB; and (**f**) SVM.

The schizophrenia dataset consists of 84 sets of EEG signals, with each set containing data from 16 channels. Figure 4 illustrates the ACC (%) for each channel across different classifiers, where a deeper color represents a higher accuracy for the respective EEG channel. For instance, Figure 4a shows the ACC results for the 16 channels when employing the DT classifier for detecting schizophrenia. The color of the P3 channel is deeper than that of the Pz channel, indicating that the accuracy of the P3 channel is higher than that of the Pz channel (P3 is 90.48%, whereas Pz is 77.38%). For further details on the four evaluation metrics, Table 2, Table 3, Table 4 and Table 5 list the results of ACC, SEN, SPE, and F1, respectively.

Analyzing the performances of the six classifiers in Table 2, Table 3, Table 4 and Table 5 reveals that all of them demonstrate impressive results, with the underlined values indicating the best performance within each classifier. Regarding the ACC, the standard deviations across all channels for LDA, NB, GAM, SVM, kNN, and DT are 4.40%, 4.62%, 4.75%, 6.16%, 6.95%, and 7.22%, respectively, with LDA, NB, and GAM showing relatively stable performance. Then, the maximum and minimum values of ACC across all channels are as follows: DT (90.48% and 61.90%), SVM (88.10% and 65.48%), LDA (85.71% and 66.67%), GAM (83.33% and 63.10%), kNN (77.38% and 57.14%), and NB (73.81% and 60.71%). These results indicate that DT, SVM, and LDA exhibit advantages in terms of accuracy. Moreover, for the other metrics, the SVM shows superior overall performance. Therefore, it can be concluded that the SVM is more suitable for the proposed method to detect schizophrenia.

**Figure 5:**
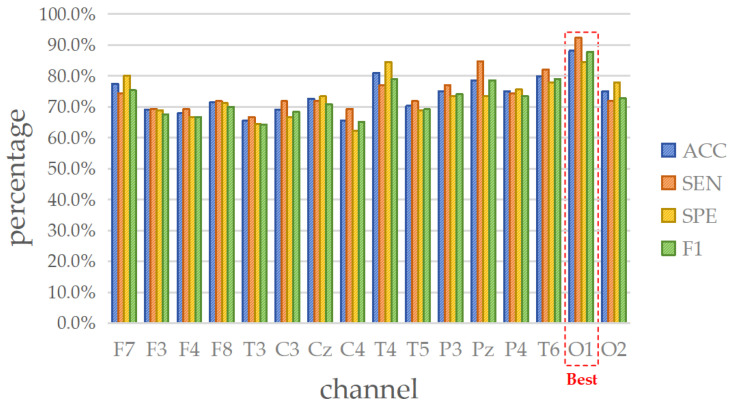
Four metrics results when using SVM on 16 channels in schizophrenia detection.

Furthermore, after investigating the suitable classifier, it is desirable to select the representative channel for minimizing data redundancy. To this end, a thorough analysis is conducted based on the four evaluation metrics. As depicted in Figure 5, the SVM classifier exhibits superior overall performance across the Pz, T4, T6, O1, and O2 channels. Particularly, the O1 channel accomplishes ACC = 88.10% (first rank), SEN = 92.31% (first rank), SPE = 84.44% (first rank), and F1 = 87.80% (first rank), demonstrating impressive performance. Hence, the O1 channel can be regarded as the representative data input for lightweight single-channel detection in the case of schizophrenia.

### 4.2. Epilepsy Results

**Figure 6:**
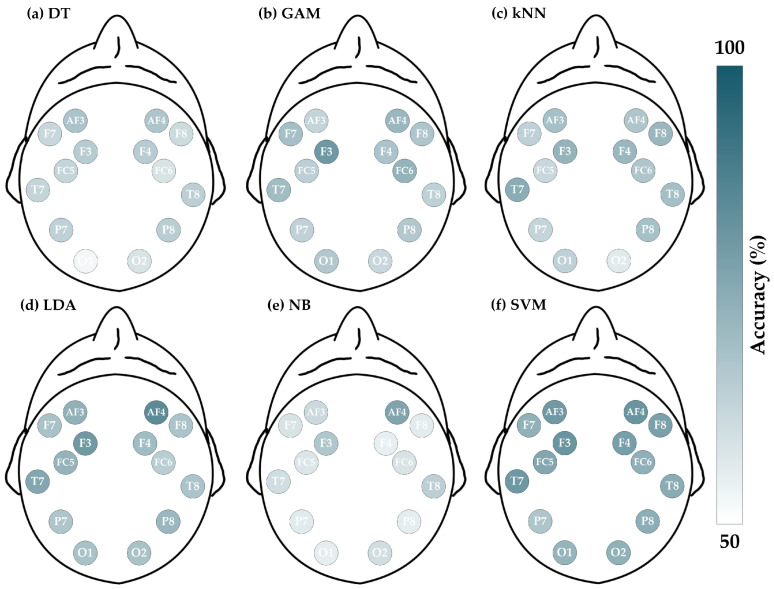
Evaluations of accuracy for epilepsy dataset by entropy-based matrix generated from 14 channels using various classifiers. The deeper the color, the greater the accuracy: (**a**) DT; (**b**) GAM; (**c**) kNN; (**d**) LDA; (**e**) NB; and (**f**) SVM.

The epilepsy dataset comprises 318 EEG recordings, each consisting of 14 channels. The ACC (%) for each channel is depicted in Figure 6, and the details of the four evaluation metrics (ACC, SEN, SPE, and F1) are listed in Table 6, Table 7, Table 8 and Table 9, respectively.

By comprehensively analyzing the results shown in Table 6, Table 7, Table 8 and Table 9, it is observed that SVM, LDA, and GAM demonstrate superior performance, while the accuracies of other classifiers are relatively lower. Besides, the standard deviations of ACC for NB, SVM, DT, kNN, GAM, and LDA are 3.27%, 3.42%, 3.61%, 3.94%, 4.03%, and 4.48%, respectively, indicating that the accuracy performance of these classifiers is stable. Meanwhile, the maximum and minimum accuracies are as follows: LDA (77.99% and 61.95%), SVM (75.47% and 63.84%), GAM (74.84% and 59.75%), kNN (69.81% and 55.35%), NB (63.52% and 54.09%), and DT (64.47% and 50.63%), where LDA, SVM, and GAM offer better output. Regarding the other metrics, the overall performance of LDA and SVM is very close. Since this paper aims to construct a detection approach suitable for multi-mental disorders, the SVM that performs remarkably in schizophrenia is chosen to be the optimal solution for epilepsy.

**Figure 7:**
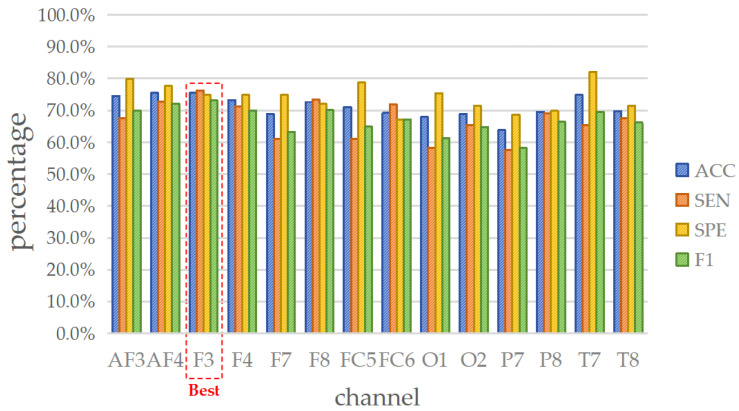
Four metrics results when using SVM on 14 channels in epilepsy detection.

Next, to determine the representative channel, all metrics of SVM across various channels are considered, as displayed in Figure 7. The assessment shows that the F3 channel offers stable satisfactory performance, where the ACC = 75.47% (1st rank), SEN = 76.26% (1st rank), SPE = 74.86% (6th rank), and F1 = 73.10% (1st rank). Therefore, it is suggested to select the F3 channel as the representative for lightweight single-channel mental disorders detection in the case of epilepsy.

### 4.3. Depression Results

**Figure 8:**
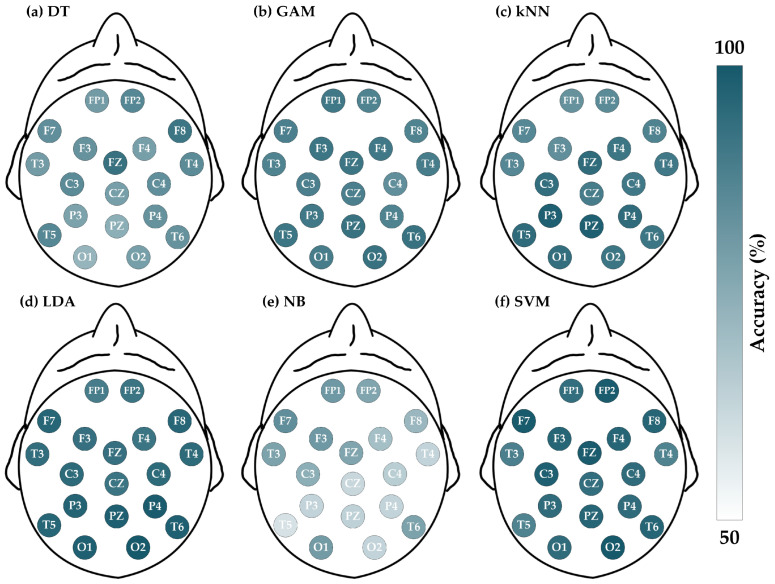
Evaluations of accuracy for depression dataset by entropy-based matrix generated from 19 channels using various classifiers. The deeper the color, the greater the accuracy: (**a**) DT; (**b**) GAM; (**c**) kNN; (**d**) LDA; (**e**) NB; and (**f**) SVM.

The depression dataset comprises 119 sets of EEG signals, each with 19 channels. Figure 8 draws the ACC (%) of all channels, and the detailed performances in terms of ACC, SEN, SPE, and F1 are contained in Table 10, Table 11, Table 12 and Table 13, respectively.

In analyzing the performance of the six classifiers in Table 10, the standard deviations of ACC for SVM, GAM, LDA, kNN, DT, and NB are 1.83%, 1.86%, 2.26%, 3.53%, 4.06%, and 6.48%, respectively, indicating good stability from them. In addition, the ACC range, with maximum and minimum values, are as follows: LDA (90.76% and 82.35%), SVM (89.92% and 84.03%), kNN (88.24% and 76.47%), GAM (84.03% and 77.31%), DT (84.03% and 68.07%), and NB (77.31% and 57.14%), where LDA, SVM, and kNN offering very similar performance. Concerning the other metrics, the performances of LDA and SVM are also very close, but SVM exhibits greater stability across all channels. As a result, the SVM demonstrates impressive accuracy and better stability, making it more suitable for depression detection in the proposed method.

**Figure 9:**
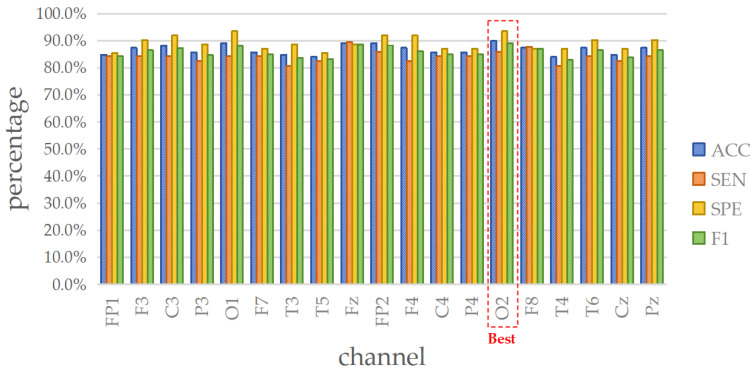
Four metrics results when using SVM on 19 channels in depression detection.

Moreover, to identify the representative channel, the four evaluation metrics of SVM are considered, as depicted in Figure 9. From the analysis, it is evident that the O2 channel generates an impressive performance, with ACC = 89.92% (first rank), SEN = 85.96% (third rank), SPE = 93.55% (first rank), and F1 = 89.09% (first rank). Hence, the O2 channel is selected as the minimal data input for lightweight single-channel mental disorders detection in the case of depression.

### 4.4. Comparative Study

To comprehensively demonstrate the advantages of this paper, a comparative study of the proposed method with recent state-of-the-art works was conducted, as outlined in Table 14. A key research gap that this study addresses is the lack of focus on single-channel selection in EEG-based mental disorder detection. While many previous studies rely on multi-channel data and complex feature extraction methods to maximize classification accuracy, few have explored the potential of single-channel EEG signals, which can greatly reduce data redundancy and improve the feasibility of portable and real-time applications. Investigating the representative channel is valuable, and it has been addressed by Cai et al. [37], who designed a prefrontal three-channel EEG signal acquisition device, achieving an accuracy rate of 86.93%. In addition, although Chen et al. [21] realized the highest accuracy of 99.16% in depression detection, the requirement for a large sample size (up to 15,360 after slicing the EEG signals) reveals this method depends on extensive training data and high computational cost. In another study by Alyas et al. [50], even with a large number of samples, the homogeneity of feature types may constrain accuracy. To characterize mental disorders more comprehensively and obtain better accuracy, several previous works [13,19,37] employed feature fusion to construct a feature matrix for classification, resulting in improved outcomes. For example, Cai et al. [37] adopted positive, negative, and neutral audio stimulation of subjects during EEG acquisition to create multimodal data sources for improving accuracy. Moreover, all of them focus on the detection of specific mental disorders. In contrast, this paper aims to design a lightweight framework that is appropriate for multi-mental disorders detection.

Compared to the previous studies, this paper aims to enhance the feature set by integrating brain rhythms and entropy features to solve the limitations of single-channel EEG signals. Although the proposed method cannot accomplish the highest accuracy in all cases, its single-channel solution effectively reduces multi-channel data redundancy and improves the portability and ease of operation of the device. Meanwhile, unlike other deep learning models [16,21,50,51] that need complex inputs, this paper employs Polynomial-SVM as the classifier due to its ability to handle non-linear relationships within the entropy-based matrix, capturing complex interactions between different brain rhythms and entropy measures, leading to superior performance compared to other classifiers. In contrast, classifiers such as kNN and DT perform worse due to their sensitivity to noise and variations in the data. Given the abstract nature of the entropy-based matrix and the non-time series format, these classifiers struggled to generalize effectively across different datasets. NB, which assumes feature independence, also shows limitations, as the features extracted from various brain rhythms are not entirely independent. This assumption led to a reduction in accuracy for models based on NB. In short, the proposed lightweight approach constructed by the entropy-based matrix has been successfully applied to the detection of at least three mental disorders, demonstrating a balance between channel number and classification accuracy. Such performances are impressive when considering fewer data sources as a concern, providing a suitable approach to multi-mental disorders detection.

### 4.5. Discussion

First, this study conducts a comprehensive analysis of the experimental mental disorders dataset and finds that the proposed framework exhibits a high degree of interpretability. Regarding the schizophrenia results, except for the best-performing O1 channel, the O2, Pz, T4, and T6 channels also demonstrate commendable performances. It suggests that EEG signals from the occipital, parietal, and temporal regions play vital roles in detecting schizophrenia. Such a finding aligns with the previous work [52], which indicates a significant positive correlation between functional connectivity deficits in the cerebellum, inferior frontal gyrus, and anterior cingulate cortex of schizophrenia patients and neurosoft markers, which are vital biological indicators of schizophrenia. In this regard, the locations of the cerebellum, inferior frontal gyrus, and anterior cingulate cortex are proximal to T4, Pz, T6, O1, and O2 channels, enhancing the clinical interpretability of the proposed method.

Second, concerning the epilepsy results, excluding the optimal F3 channel, the AF3, AF4, and F4 channels also provide good detection performance. Such high-performing channels are mainly located in the frontal lobe region. Considering that epilepsy seizures are normally caused by abnormal discharges in the brain, the entropy-based matrix in this study helps to characterize abnormal brain rhythms and demonstrates certain reliability, revealing that the seizures in this dataset exhibit correlations with the frontal and prefrontal regions. Particularly, several previous studies [53,54,55] have mentioned that during seizures, discharges from the temporal lobe, hippocampus, and amygdala typically propagate along specific neural pathways to the frontal lobe, potentially impairing its functions. This finding is also consistent with the experimental results obtained in this paper.

Lastly, as for the depression results, the O1 channel offers remarkable performances based on the four evaluation metrics. Meanwhile, the O2 channel also performs well. In addition to the occipital region, the average accuracy of the frontal region (F3, F7, F4, F8, and Fz) is 87.39%, while the average accuracy of the parietal region (P3, P4, and Pz) is 86.27%, slightly inferior to that of the frontal region. In both the frontal and parietal regions, the accuracy of Fz (89.08%) and Pz (87.39%) is higher than their averages. The previous work [56] claimed that the brain mechanisms underlying depression are mainly concentrated in the midline region of the brain. It is meaningful that the locations of the Fz, Pz, O1, and O2 channels are situated in and around the midline of the brain, further confirming the experimental results of this study and advancing insights into the underlying mechanisms and pathological states of depression.

## 5. Conclusions

In this paper, a lightweight EEG-based multi-mental disorders detection method is proposed. It first applies DTW to decompose EEG signals into five brain rhythms, followed by extracting entropy features from these rhythms, which are then gathered into an entropy-based matrix. After that, conventional machine learning classifiers are employed to train and test the entropy-based matrix. The method validation demonstrates the impressive performances using three public EEG datasets in schizophrenia, epilepsy, and depression, achieving satisfying accuracies of 88.10%, 75.47%, and 89.92%, respectively. In addition, the results not only confirm the robustness of Polynomial-SVM in detecting multi-mental disorders but also help entropy features that characterize such conditions, enhancing the interpretability of the EEG signals. Furthermore, the selected representative channel can support the detection through the single-channel solution, which provides insights into the underlying mechanisms and pathological states of brain functions in terms of mental disorders. Consequently, the proposed method holds the potential for embedding into portable devices that assist in the early detection of mental disorders, which help in timely intervention and prevent the adverse consequences of delayed diagnosis and treatment.

However, there are several limitations to this approach. First, the reliance on EEG as a single modality may limit the richness of information compared to multimodal approaches, which can integrate diverse data types such as audio and facial expression, potentially limiting the method’s ability to capture a wider range of brain activities. Second, while the proposed method achieves reasonable classification accuracy, the recognition rates are relatively lower compared to certain deep learning models, which can process more complex data inputs. Therefore, several related data compression and feature fusion methods [57,58,59,60] will be investigated in the future. In addition, the EEG cases of other mental disorders, such as autism, anxiety, dementia, etc., will be analyzed, facilitating the diagnosis in more healthcare applications through this lightweight single-channel solution.

## Figures and Tables

**Table 1:** Key information of three EEG mental disorders datasets.

Dataset	Schizophrenia	Epilepsy	Depression
Number of participants (Healthy/Patient)	84 (39/45)	318 (139/179)	119 (57/62)
Number of EEG channels	16	14	19
Time duration	1-min	approximately five minutes	300 s
Sampling rate	128 Hz	128 Hz	256 Hz

**Table 2:** Accuracy (ACC) results of six classifiers on 16 channels in schizophrenia detection.

	DT	GAM	kNN	LDA	NB	SVM
F7	71.43%	76.19%	77.38%	71.43%	60.71%	77.38%
F3	72.62%	67.86%	73.81%	73.81%	63.10%	69.05%
F4	61.90%	70.24%	72.62%	69.05%	71.43%	67.86%
F8	69.05%	63.10%	65.48%	77.38%	60.71%	71.43%
T3	77.38%	71.43%	67.86%	66.67%	61.90%	65.48%
C3	72.62%	70.24%	75.00%	72.62%	67.86%	69.05%
Cz	80.95%	75.00%	57.14%	76.19%	73.81%	72.62%
C4	79.76%	83.33%	58.33%	73.81%	63.10%	65.48%
T4	73.81%	70.24%	60.71%	75.00%	64.29%	80.95%
T5	69.05%	69.05%	61.90%	69.05%	65.48%	70.24%
P3	90.48%	73.81%	60.71%	73.81%	67.86%	75.00%
Pz	77.38%	73.81%	59.52%	85.71%	73.81%	78.57%
P4	85.71%	75.00%	65.48%	75.00%	71.43%	75.00%
T6	82.14%	69.05%	59.52%	71.43%	70.24%	79.76%
O1	69.05%	73.81%	63.10%	77.38%	72.62%	88.10%
O2	72.62%	65.48%	76.19%	71.43%	67.86%	75.00%

**Table 3:** Sensitivity (SEN) results of six classifiers on 16 channels in schizophrenia detection.

	DT	GAM	kNN	LDA	NB	SVM
F7	61.54%	71.79%	79.49%	66.67%	66.67%	74.36%
F3	56.41%	69.23%	76.92%	74.36%	66.67%	69.23%
F4	56.41%	74.36%	76.92%	66.67%	79.49%	69.23%
F8	64.10%	56.41%	61.54%	74.36%	56.41%	71.79%
T3	76.92%	66.67%	71.79%	71.79%	53.85%	66.67%
C3	69.23%	64.10%	76.92%	71.79%	71.79%	71.79%
Cz	71.79%	76.92%	58.97%	76.92%	76.92%	71.79%
C4	74.36%	79.49%	58.97%	76.92%	64.10%	69.23%
T4	69.23%	64.10%	58.97%	74.36%	64.10%	76.92%
T5	64.10%	56.41%	56.41%	69.23%	58.97%	71.79%
P3	89.74%	71.79%	61.54%	71.79%	69.23%	76.92%
Pz	76.92%	76.92%	58.97%	89.74%	74.36%	84.62%
P4	84.62%	69.23%	64.10%	79.49%	74.36%	74.36%
T6	87.18%	66.67%	56.41%	74.36%	76.92%	82.05%
O1	74.36%	71.79%	56.41%	76.92%	74.36%	92.31%
O2	74.36%	58.97%	76.92%	74.36%	74.36%	71.79%

**Table 4:** Specificity (SPE) results of six classifiers on 16 channels in schizophrenia detection.

	DT	GAM	kNN	LDA	NB	SVM
F7	80.00%	80.00%	75.56%	75.56%	55.56%	80.00%
F3	86.67%	66.67%	71.11%	73.33%	60.00%	68.89%
F4	66.67%	66.67%	68.89%	71.11%	64.44%	66.67%
F8	73.33%	68.89%	68.89%	80.00%	64.44%	71.11%
T3	77.78%	75.56%	64.44%	62.22%	68.89%	64.44%
C3	75.56%	75.56%	73.33%	73.33%	64.44%	66.67%
Cz	88.89%	73.33%	55.56%	75.56%	71.11%	73.33%
C4	84.44%	86.67%	57.78%	71.11%	62.22%	62.22%
T4	77.78%	75.56%	62.22%	75.56%	64.44%	84.44%
T5	73.33%	80.00%	66.67%	68.89%	71.11%	68.89%
P3	91.11%	75.56%	60.00%	75.56%	66.67%	73.33%
Pz	77.78%	71.11%	60.00%	82.22%	73.33%	73.33%
P4	86.67%	80.00%	66.67%	71.11%	68.89%	75.56%
T6	77.78%	71.11%	62.22%	68.89%	64.44%	77.78%
O1	64.44%	75.56%	68.89%	77.78%	71.11%	84.44%
O2	71.11%	71.11%	75.56%	68.89%	62.22%	77.78%

**Table 5:** F1-score (F1) results of six classifiers on 16 channels in schizophrenia detection.

	DT	GAM	kNN	LDA	NB	SVM
F7	66.67%	73.68%	76.54%	68.42%	61.18%	75.32%
F3	65.67%	66.67%	73.17%	72.50%	62.65%	67.50%
F4	57.89%	69.88%	72.29%	66.67%	72.09%	66.67%
F8	65.79%	58.67%	62.34%	75.32%	57.14%	70.00%
T3	75.95%	68.42%	67.47%	66.67%	56.76%	64.20%
C3	70.13%	66.67%	74.07%	70.89%	67.47%	68.29%
Cz	77.78%	74.07%	56.10%	75.00%	73.17%	70.89%
C4	77.33%	81.58%	56.79%	73.17%	61.73%	65.06%
T4	71.05%	66.67%	58.23%	73.42%	62.50%	78.95%
T5	65.79%	62.86%	57.89%	67.50%	61.33%	69.14%
P3	89.74%	71.79%	59.26%	71.79%	66.67%	74.07%
Pz	75.95%	73.17%	57.50%	85.37%	72.50%	78.57%
P4	84.62%	72.00%	63.29%	74.70%	70.73%	73.42%
T6	81.93%	66.67%	56.41%	70.73%	70.59%	79.01%
O1	69.05%	71.79%	58.67%	75.95%	71.60%	87.80%
O2	71.60%	61.33%	75.00%	70.73%	68.24%	72.73%

**Table 6:** Accuracy (ACC) results of six classifiers on 14 channels in epilepsy detection.

	DT	GAM	kNN	LDA	NB	SVM
AF3	64.47%	60.06%	65.09%	68.87%	59.12%	74.53%
AF4	64.15%	67.30%	64.15%	77.99%	63.52%	75.47%
F3	62.58%	74.84%	68.24%	74.53%	63.52%	75.47%
F4	62.26%	64.47%	67.30%	66.04%	54.09%	73.27%
F7	59.43%	65.72%	61.01%	64.78%	56.60%	68.87%
F8	58.81%	63.84%	67.30%	64.47%	54.72%	72.64%
FC5	60.06%	61.64%	59.75%	68.24%	55.97%	71.07%
FC6	56.60%	68.24%	64.15%	61.95%	56.92%	69.18%
O1	50.63%	63.21%	61.32%	64.78%	54.40%	67.92%
O2	56.60%	59.75%	55.35%	64.78%	58.18%	68.87%
P7	61.32%	60.69%	60.06%	63.84%	55.35%	63.84%
P8	61.95%	63.21%	65.09%	67.30%	54.40%	69.50%
T7	60.06%	66.04%	69.81%	71.07%	57.86%	74.84%
T8	61.64%	61.32%	65.41%	64.47%	61.64%	69.81%

**Table 7:** Sensitivity (SEN) results of six classifiers on 14 channels in epilepsy detection.

	DT	GAM	kNN	LDA	NB	SVM
AF3	53.96%	53.24%	62.59%	64.75%	75.54%	67.63%
AF4	63.31%	62.59%	52.52%	74.10%	82.01%	72.66%
F3	56.12%	72.66%	72.66%	75.54%	97.12%	76.26%
F4	58.99%	57.55%	67.63%	61.87%	62.59%	71.22%
F7	55.40%	61.15%	53.96%	53.96%	67.63%	61.15%
F8	44.60%	58.27%	64.03%	58.27%	69.78%	73.38%
FC5	52.52%	55.40%	52.52%	58.99%	41.01%	61.15%
FC6	48.20%	63.31%	59.71%	58.99%	74.82%	71.94%
O1	41.73%	57.55%	58.99%	47.48%	53.24%	58.27%
O2	55.40%	53.96%	54.68%	56.12%	65.47%	65.47%
P7	50.36%	56.12%	51.80%	48.92%	33.81%	57.55%
P8	61.15%	59.71%	64.03%	61.87%	71.94%	69.06%
T7	56.12%	59.71%	65.47%	59.71%	44.60%	65.47%
T8	52.52%	52.52%	64.75%	58.99%	72.66%	67.63%

**Table 8:** Specificity (SPE) results of six classifiers on 14 channels in epilepsy detection.

	DT	GAM	kNN	LDA	NB	SVM
AF3	72.63%	65.36%	67.04%	72.07%	46.37%	79.89%
AF4	64.80%	70.95%	73.18%	81.01%	49.16%	77.65%
F3	67.60%	76.54%	64.80%	73.74%	37.43%	74.86%
F4	64.80%	69.83%	67.04%	69.27%	47.49%	74.86%
F7	62.57%	69.27%	66.48%	73.18%	48.04%	74.86%
F8	69.83%	68.16%	69.83%	69.27%	43.02%	72.07%
FC5	65.92%	66.48%	65.36%	75.42%	67.60%	78.77%
FC6	63.13%	72.07%	67.60%	64.25%	43.02%	67.04%
O1	57.54%	67.60%	63.13%	78.21%	55.31%	75.42%
O2	57.54%	64.25%	55.87%	71.51%	52.51%	71.51%
P7	69.83%	64.25%	66.48%	75.42%	72.07%	68.72%
P8	62.57%	65.92%	65.92%	71.51%	40.78%	69.83%
T7	63.13%	70.95%	73.18%	79.89%	68.16%	82.12%
T8	68.72%	68.16%	65.92%	68.72%	53.07%	71.51%

**Table 9:** F1-score (F1) results of six classifiers on 14 channels in epilepsy detection.

	DT	GAM	kNN	LDA	NB	SVM
AF3	57.03%	53.82%	61.05%	64.52%	61.76%	69.89%
AF4	60.69%	62.59%	56.15%	74.64%	66.28%	72.14%
F3	56.73%	71.63%	66.67%	72.16%	69.95%	73.10%
F4	57.75%	58.61%	64.38%	61.43%	54.38%	69.96%
F7	54.42%	60.93%	54.74%	57.25%	57.67%	63.20%
F8	48.63%	58.48%	63.12%	58.91%	57.40%	70.10%
FC5	53.48%	55.80%	53.28%	61.89%	44.88%	64.89%
FC6	49.26%	63.54%	59.29%	57.54%	60.29%	67.11%
O1	42.49%	57.76%	57.14%	54.10%	50.51%	61.36%
O2	52.74%	53.96%	51.70%	58.21%	57.78%	64.77%
P7	53.23%	55.52%	53.14%	54.18%	39.83%	58.18%
P8	58.42%	58.66%	61.59%	62.32%	57.97%	66.44%
T7	55.12%	60.58%	65.47%	64.34%	48.06%	69.47%
T8	54.48%	54.28%	62.07%	59.21%	62.35%	66.20%

**Table 10:** Accuracy (ACC) results of six classifiers on 19 channels in depression detection.

	DT	GAM	kNN	LDA	NB	SVM
FP1	73.95%	82.35%	76.47%	82.35%	74.79%	84.87%
F3	76.47%	83.19%	77.31%	84.87%	74.79%	87.39%
C3	78.15%	80.67%	84.87%	86.55%	69.75%	88.24%
P3	72.27%	82.35%	88.24%	88.24%	60.50%	85.71%
O1	68.07%	81.51%	84.87%	89.08%	73.95%	89.08%
F7	77.31%	80.67%	78.99%	87.39%	77.31%	85.71%
T3	73.95%	79.83%	78.99%	85.71%	72.27%	84.87%
T5	78.99%	83.19%	85.71%	87.39%	57.14%	84.03%
Fz	84.03%	83.19%	85.71%	84.87%	71.43%	89.08%
FP2	78.99%	79.83%	78.15%	84.87%	71.43%	89.08%
F4	73.11%	82.35%	83.19%	84.03%	65.55%	87.39%
C4	77.31%	77.31%	82.35%	86.55%	62.18%	85.71%
P4	76.47%	84.03%	85.71%	89.92%	60.50%	85.71%
O2	73.11%	84.03%	83.19%	90.76%	60.50%	89.92%
F8	83.19%	79.83%	79.83%	87.39%	67.23%	87.39%
T4	78.15%	81.51%	82.35%	86.55%	60.50%	84.03%
T6	76.47%	84.03%	83.19%	89.08%	72.27%	87.39%
Cz	73.11%	80.67%	81.51%	84.03%	59.66%	84.87%
Pz	69.75%	84.03%	88.24%	89.08%	61.34%	87.39%

**Table 11:** Sensitivity (SEN) results of six classifiers on 19 channels in depression detection.

	DT	GAM	kNN	LDA	NB	SVM
FP1	68.42%	77.19%	75.44%	78.95%	66.67%	84.21%
F3	75.44%	84.21%	77.19%	78.95%	73.68%	84.21%
C3	78.95%	75.44%	87.72%	80.70%	91.23%	84.21%
P3	70.18%	84.21%	91.23%	85.96%	89.47%	82.46%
O1	66.67%	80.70%	85.96%	84.21%	64.91%	84.21%
F7	75.44%	80.70%	78.95%	84.21%	71.93%	84.21%
T3	68.42%	78.95%	77.19%	75.44%	73.68%	80.70%
T5	78.95%	85.96%	92.98%	80.70%	91.23%	82.46%
Fz	82.46%	80.70%	89.47%	80.70%	87.72%	89.47%
FP2	80.70%	78.95%	82.46%	80.70%	85.96%	85.96%
F4	78.95%	84.21%	82.46%	80.70%	84.21%	82.46%
C4	78.95%	77.19%	87.72%	85.96%	94.74%	84.21%
P4	80.70%	89.47%	91.23%	85.96%	94.74%	84.21%
O2	70.18%	85.96%	84.21%	87.72%	94.74%	85.96%
F8	87.72%	82.46%	80.70%	82.46%	89.47%	87.72%
T4	73.68%	82.46%	84.21%	82.46%	94.74%	80.70%
T6	80.70%	82.46%	80.70%	84.21%	84.21%	84.21%
Cz	71.93%	84.21%	84.21%	82.46%	96.49%	82.46%
Pz	71.93%	84.21%	89.47%	87.72%	94.74%	84.21%

**Table 12:** Specificity (SPE) results of six classifiers on 19 channels in depression detection.

	DT	GAM	kNN	LDA	NB	SVM
FP1	79.03%	87.10%	77.42%	85.48%	82.26%	85.48%
F3	77.42%	82.26%	77.42%	90.32%	75.81%	90.32%
C3	77.42%	85.48%	82.26%	91.94%	50.00%	91.94%
P3	74.19%	80.65%	85.48%	90.32%	33.87%	88.71%
O1	69.35%	82.26%	83.87%	93.55%	82.26%	93.55%
F7	79.03%	80.65%	79.03%	90.32%	82.26%	87.10%
T3	79.03%	80.65%	80.65%	95.16%	70.97%	88.71%
T5	79.03%	80.65%	79.03%	93.55%	25.81%	85.48%
Fz	85.48%	85.48%	82.26%	88.71%	56.45%	88.71%
FP2	77.42%	80.65%	74.19%	88.71%	58.06%	91.94%
F4	67.74%	80.65%	83.87%	87.10%	48.39%	91.94%
C4	75.81%	77.42%	77.42%	87.10%	32.26%	87.10%
P4	72.58%	79.03%	80.65%	93.55%	29.03%	87.10%
O2	75.81%	82.26%	82.26%	93.55%	29.03%	93.55%
F8	79.03%	77.42%	79.03%	91.94%	46.77%	87.10%
T4	82.26%	80.65%	80.65%	90.32%	29.03%	87.10%
T6	72.58%	85.48%	85.48%	93.55%	61.29%	90.32%
Cz	74.19%	77.42%	79.03%	85.48%	25.81%	87.10%
Pz	67.74%	83.87%	87.10%	90.32%	30.65%	90.32%

**Table 13:** F1-score (F1) results of six classifiers on 19 channels in depression detection.

	DT	GAM	kNN	LDA	NB	SVM
FP1	71.56%	80.73%	75.44%	81.08%	71.70%	84.21%
F3	75.44%	82.76%	76.52%	83.33%	73.68%	86.49%
C3	77.59%	78.90%	84.75%	85.19%	74.29%	87.27%
P3	70.80%	82.05%	88.14%	87.50%	68.46%	84.68%
O1	66.67%	80.70%	84.48%	88.07%	70.48%	88.07%
F7	76.11%	80.00%	78.26%	86.49%	75.23%	84.96%
T3	71.56%	78.95%	77.88%	83.50%	71.79%	83.64%
T5	78.26%	83.05%	86.18%	85.98%	67.10%	83.19%
Fz	83.19%	82.14%	85.71%	83.64%	74.63%	88.70%
FP2	78.63%	78.95%	78.33%	83.64%	74.24%	88.29%
F4	73.77%	82.05%	82.46%	82.88%	70.07%	86.24%
C4	76.92%	76.52%	82.64%	85.96%	70.59%	84.96%
P4	76.67%	84.30%	85.95%	89.09%	69.68%	84.96%
O2	71.43%	83.76%	82.76%	90.09%	69.68%	89.09%
F8	83.33%	79.66%	79.31%	86.24%	72.34%	86.96%
T4	76.36%	81.03%	82.05%	85.45%	69.68%	82.88%
T6	76.67%	83.19%	82.14%	88.07%	74.42%	86.49%
Cz	71.93%	80.67%	81.36%	83.19%	69.62%	83.93%
Pz	69.49%	83.48%	87.93%	88.50%	70.13%	86.49%

**Table 14:** A comparative study with recent works.

Work	Number of Channels	Number of Subjects	Feature	Classifier	Accuracy (%)		
					Schizophrenia	Epilepsy	Depression
Movahed et al. [13]	19	119	Statistical, spectral, wavelet, functional connectivity, and non-linear features	Radial Basis Function (RBF)-SVM	/	/	90.70
Qiao et al. [14]	20	64	EEG feature map	TanhReLU-based CNN	/	/	98.59
Gupta et al. [15]	3	55	EEG and audio multimodal data	Bi-LSTM	/	/	99.90
Hu et al. [16]	19	/	Log-amplitude characteristics of the four seizure classes	IGGCN	/	91.80	/
Kumar et al. [19]	16	84	HLV and SLBP features	Adaptive Boosting (AdaBoost)	83.33	/	/
Chen et al. [21]	19	119	Two-dimensional matrices with different frequency domains and electrode position	CNN+LSTM	/	/	99.16
Cai et al. [37]	3	119	Both linear and non-linear features	kNN	/	/	86.93
Alyas et al. [50]	14	318	Statistical features	Extreme Gradient Boosting (XGBoost)	/	79.45	/
Hussain et al. [51]	16	84	Six-second EEG trial divided into three-second windows	1D-CNN with majority voting	99.88	/	/
This study	1	84/318/119	Entropy-based matrix from brain rhythms	Polynomial-SVM	88.10	75.47	89.92

## Data Availability

The datasets generated and/or analyzed during the current study are available at https://github.com/75Fleven/CODE.git (accessed on 31 August 2024).
